# There Is No Influence of Egg Size on Sex Allocation in Arrhenotokous Lineages of *Thrips tabaci* Lindeman

**DOI:** 10.3390/insects13050408

**Published:** 2022-04-24

**Authors:** Saranda Musa, Márta Ladányi, József Fail

**Affiliations:** 1 Department of Entomology, Institute of Plant Protection, Hungarian University of Agriculture and Life Sciences, Menesi út 44, 1118 Budapest, Hungary; sarandamusa1@gmail.com; 2 Department of Plant Protection, Faculty of Agriculture and Veterinary, University of Prishtina, 10000 Prishtina, Kosovo; 3 Department of Applied Statistics, Institute of Mathematics and Basic Science, Hungarian University of Agriculture and Life Sciences, Villanyi út 29-43, 1118 Budapest, Hungary; ladanyi.marta@uni-mate.hu

**Keywords:** egg size, offspring fitness, sex allocation, *Thrips tabaci*, Thripidae

## Abstract

**Simple Summary:**

How parents control the sex of their offspring greatly differs in the animal kingdom. Two lineages in the *Thrips tabaci* Lindeman cryptic species complex exhibit arrhenotokous haplodiploidy, which enables parents to influence the sex of offspring by different fertilization mechanisms. In two other haplodiploid species, sex allocation is mediated by egg size. Contrary to the two haplodiploid arthropods with an egg-size-mediated sex allocation mechanism, our study proves that a different mechanism that is independent of egg size regulates sex allocation in *T. tabaci*. The results presented in this paper raise intriguing questions regarding the evolutionary forces driving egg size and sex allocation in haplodiploids. In addition, our results indicate a significant reduction in egg size with increasing maternal age that cannot be attributed to the resource depletion hypothesis.

**Abstract:**

In two haplodiploid species, sex allocation in young arrhenotokous females is mediated by egg size. We tested if sex allocation is mediated by egg size in two arrhenotokous lineages of the haplodiploid species complex of *T. tabaci*: L1 and T. We measured the size of eggs produced by very young mothers, 3–5-day-old mothers (just like in the case of *Tetranychus urticae*) and 7–10-day-old mothers (as in *Pezothrips kellyanus*). Moreover, we measured the size of eggs oviposited by mothers in their entire lifespan. We found that in *T. tabaci,* sex allocation is not mediated by egg size. Egg size and gender were independent of maternal age in the L1 lineage, whilst in the T lineage, the observed egg size difference between males and females was only present in the progeny of young females (7–10-day-old mothers). Furthermore, we found that male eggs produced by mated mothers were larger than those produced by virgin mothers in the L1 lineage, but in the T lineage, there were no differences in the size of male eggs produced by mated and virgin mothers. Our results indicate that these two subspecies have different resource allocation strategies in response to maternal mating status.

## 1. Introduction

Studies of sex allocation have been the most intriguing and productive areas in evolutionary biology [1]. There has been theoretical and empirical research supporting sex allocation. The theory predicts how individuals should divide their investment of resources between male and female offspring in response to environmental conditions, and there are an enormous number of supporting empirical studies [1,2,3,4,5]. The theory of sex allocation states that, under certain circumstances, the marginal fitness benefit of allocating resources to male or female reproduction differs when selecting for biased sex allocation [6,7].

The biased sex ratio in progeny is the most obvious way to adjust sex allocation [8]. This aspect of sex allocation has been widely studied in a variety of both invertebrate and vertebrate organisms [9,10]. According to several authors, parental investment in eggs can affect offspring fitness [11,12]. Offspring that benefit from inheriting more resources from their parents have greater fitness, and one of the most widely used predictors of such investment is egg size. However, the adjustment of egg size depending on the sex of the embryo has been less investigated. These sex-biased parental strategies have been demonstrated in birds [13,14,15,16] and lizards [17]. Haplodiploidy is a sex-determination system that enables females to control the sex of their offspring through fertilization control. That is, females are diploid and develop from fertilized eggs while males are haploid and develop from unfertilized eggs [1]; evidence is the rearing of only male progeny from eggs laid by virgin females. With arrhenotokous haplodiploid sex determination, females control the sex of their offspring by regulating the access of sperm to the egg. Following insemination, sperm is stored in a spermatheca. Evidence for the ability of females to control the sex ratio in thrips by selective fertilization was provided by Priesner [18], who described the thrips spermatheca and associated musculature. Males can also influence sex allocation in different ways [19]. First, the ability of males to fertilize eggs is variable, with some males producing sperm that is unable to successfully fertilize eggs. Second, the incompatibility of the paternal and maternal genomes leads to the embryonic death of female offspring. Third, males can attempt to increase daughter production because males can only pass genes to their daughters [20]. In haplodiploid arthropods, egg-size-mediated sex allocation studies have been reported for the two-spotted spider mite *Tetranychus urticae* Koch (Prostigmata: Tetranychidae) [21] and Kelly’s citrus thrips *Pezothrips kellyanus* (Bagnall) (Thysanoptera: Thripidae) [22]. In these two species, egg size determines not only the larval size, juvenile survival, and adult size but also the probability of fertilization, with female (fertilized) eggs being larger than male (unfertilized) eggs. Since there are no further reports available for other haplodiploid arthropods, we intended to study this mechanism in the two arrhenotokous lineages of onion thrips.

Onion thrips, *Thrips tabaci* Lindeman (Thysanoptera: Thripidae), has received considerable attention due to its cryptic life habitat and mode of reproduction. The pest status of onion thrips can be attributed to its polyphagous nature, high reproductive capacity, short generation time, transmission of plant viruses, and development of resistance to insecticides [23,24]. It has been one of the most intensively studied thrips species, though some of the biological aspects of this species remain unknown [25]. DNA sequences of the mitochondrial COI gene have confirmed that *T. tabaci* is a species complex, and it has been divided into three lineages: L1, L2 (leek-associated), and T (tobacco-associated) [26]. L1 leek-associated and T tobacco-associated lineages have arrhenotokous reproduction [27,28], while the L2 leek-associated lineage has thelytokous reproduction [29]. Despite the great potential of thrips for the study of sex allocation, data regarding sex allocation for this order is rather sparse.

*T. tabaci* has an arrhenotokous haplodiploid sex-determination system that is common in the order Thysanoptera [30]. Virgin arrhenotokous females produce only haploid male eggs, while mated arrhenotokous females produce diploid female and haploid male eggs. Different investigations into *T. tabaci* reproductive modes have been carried out so far. According to Nault et al. [31], neither high temperatures nor *Wolbachia* bacteria have any effect on sex ratios in either thelytokous or male-producing populations.

The aim of our study was to investigate the influence of egg size on sex allocation in the two arrhenotokous lineages of the *T. tabaci* cryptic species complex. Prior to this, we tested the effect of mating and egg size on hatching probability. Furthermore, we investigated the effect of maternal mating status on the size of male eggs produced by virgin and mated mothers, as well as the effect of maternal age on egg size regardless of the offspring sex.

## 2. Materials and Methods

**Figure 1:**
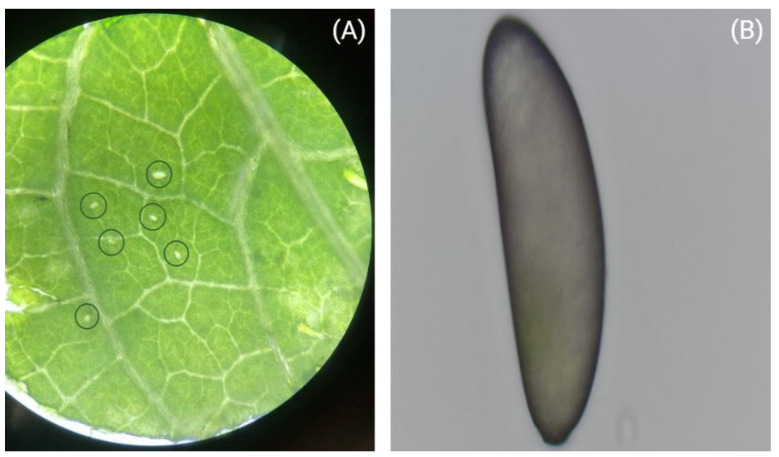
*Thrips tabaci* eggs inserted into a bean leaf (**A**) and an egg excavated from plant tissue (**B**).

The L1 and T arrhenotokous onion thrips colonies were established in 2014 at the Department of Entomology, Institute of Plant Protection. The lineages of *T. tabaci* were identified with molecular identification [28]. As the eggs of terebrantian thrips are laid into the leaf tissue (Figure 1A), bean leaves (*Phaseolus vulgaris* L.) were used for all treatments to facilitate the careful excavation of eggs (Figure 1B), since bean leaves are soft and provide the opportunity to handle the eggs easily. All experiments were performed in a growth chamber (Peltier-cooled incubator IPP260plus, Memmert GmbH + Co.KG, Schwabach, Germany) under controlled conditions at 23 °C with a light and dark photoperiod regime of L16: D8 h.

### 2.1. Egg Collection from Virgin and Mated Females of L1 and T Lineages

In order to measure the size of eggs produced by virgin and mated females of the L1 and T lineages of *T. tabaci*, pupae were collected from the stock colonies and separately isolated in order to ensure the virginity of the females. Pupae were observed every twelve hours in order to record the time of adult emergence. In total, 77 pupae of the L1 lineage and 69 pupae of the T lineages were collected and individually reared in a 2 mL Eppendorf tube on bean (*P. vulgaris*) leaf discs less than 1 cm in diameter until they reached the adult stage. From 77 pupae collected from the L1 colony, 42 females and 15 males emerged. In the case of the T lineage, 45 females and 21 male adults emerged. Right after adult emergence, 16 females from the L1 lineage and 19 females from the T lineage were paired with the emerging males, one female and one male in each Eppendorf tube containing a bean leaf disk. Females and males were kept together for 48 h in order to ensure mating. Then, males were removed and preserved in 96% ethanol for DNA-based identification. Meanwhile, the remaining females, of which there were 26 for each lineage, were kept as virgins. All the virgin and mated females were transferred to new bean leaf disks for oviposition throughout their lifespan. The bean leaf disks were changed every twelve hours until the given female died.

Eggs that were laid into a bean leaf disk were carefully excavated with a needle under a stereo microscope (Alpha, NSZ-606, Novel optics, Ningbo Yongxin, Ningbo, China). Subsequently, eggs were placed on a microscopic slide under a calibrated compound light microscope (LEICA DM LB, Leica Microsystem GmBH, Wetzlar, Germany) with an ocular graticule. Two dimensions (width and length) of an egg were measured under 600× magnification. After the measurement, the eggs were carefully placed back into the tube with the help of the needle, each egg being individually placed on a leaf disk to facilitate hatching and consequent juvenile development. Eggs were characterized by the mother ID, mother age, time of oviposition, size, hatching date, and (later on) sex when it became detectable (first stage larvae of mated females were slide-mounted and sexed according to the procedure of Vierbergen et al. [32]).

### 2.2. Egg Volume

The formula below for calculating the volume of eggs was taken from the work of Church et al. [33], which we think is more tailored for the shape of *T. tabaci* eggs than the formula for the prolate ellipsoid shape used by Katlav et al. [22]. Using the formula, the volume was calculated from two measured dimensions, namely length and width, assuming that thickness and width are equal:

$$V=\frac{width*thickness*length*\pi }{6}=\frac{width^{2}*length*\pi }{6}$$

### 2.3. Effect of Mating and Egg Size on the Hatching Probability of the Eggs

To test whether egg size is a predictor of energy content in the L1 and T arrhenotokous lineages, we assessed the effect of mating and egg size on the hatching probability of the eggs in the progeny of virgin and mated mothers. Egg hatchability was recorded as hatched and unhatched eggs for all the eggs that were laid during the entire lifespan of the mothers. We compared the egg hatchability rates between virgin and mated mothers of the L1 and T lineages using the chi-squared test. Using the one-way random block design MANOVA, we compared the size of eggs (width, length, and volume) laid by virgin and mated mothers of the L1 and T lineages according to their hatchability (hatched and did not hatch), with the factor of ‘hatchability’ and mother ID as a block.

### 2.4. Comparison of Male and Female Egg Size Produced by Mated Mothers of the L1 and T Lineages

To quantify maternal investment in eggs, we compared the size of eggs in male and female offspring produced by mated mothers during their entire lifespan. In two studied species, *T. urticae* and P. *kellyanus*, egg size and gender were found to be correlated with the egg mass produced by young arrhenotokous females. Corresponding to these approaches, we compared the size of male and female eggs in different age groups of mothers: 3–5-day-old mothers (just like in *T. urticae* [21]), 7–10-day-old mothers (similar to *P. kellyanus* [22]), and above-10-day-old mothers. The analysis of the egg size (width, length, and volume) of male and female offspring produced by mated mothers of the L1 and T lineages was performed using one-way multivariate analysis of variance (MANOVA) with the factor of ‘gender’. We carried out the same analysis for all age groups.

### 2.5. Testing the Model of Egg Size and Sex Allocation

**Figure 2:**
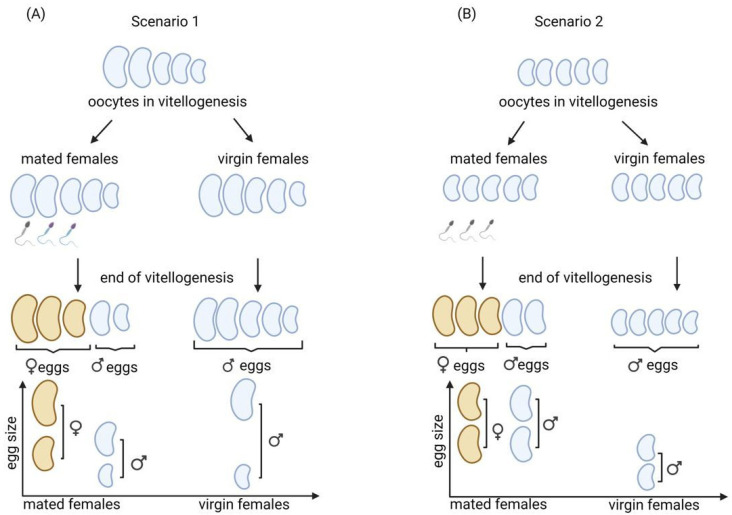
Hypothetical mechanisms of sex-specific egg size in haplodiploid arrhenotokous lineages of *T. tabaci*. (**A**) Scenario 1: Differences in egg size exist before fertilization, and egg size determines the probability of an egg being fertilized. Under this scenario, the egg size range between virgin and mated mothers should be the same. (**B**) Scenario 2: Egg size is equal in virgin and mated mothers prior to fertilization, and mating increases the egg size in general. Straight vertical lines represent egg size ranges. Fertilized and unfertilized eggs are presented in brown and blue, respectively.

We tested whether an asymmetric allocation by mothers in the male and female eggs could occur before fertilization and whether egg size determines the probability of an egg being fertilized (a female fertilized egg being larger than a male unfertilized egg) (Figure 2A, scenario 1). Under this scenario, the range of egg size should be similar between virgin and mated mothers. Moreover, any egg size sex-specific differences would result from the selective fertilization of larger eggs, leading to the larger egg size of males produced by virgin mothers than by mated mothers.

Second, we tested whether mating affects the female resource allocation strategy if eggs draw more resources once they are fertilized, which is also possible because eggs in the majority of insects, including thrips, are fertilized just before the end of vitellogenesis [34] (Figure 2B, scenario 2). Under this scenario, the size of the eggs is expected to be larger among eggs from mated mothers than among virgins, and mating should increase the egg size in general, leading to an equal egg size in males and females produced by mated mothers and, therefore, a smaller egg size in males produced by virgin mothers.

To discriminate between these two scenarios, we compared the mean and the distribution of the egg size produced by virgin and mated mothers. The analysis of egg size (width, length, and volume) produced by virgin and mated mothers of the L1 and T lineages was performed for the entire lifespan of the mothers and age groups using one-way MANOVA, where the factor was ‘mating status’.

### 2.6. Comparison of Male Egg Size Produced by Virgin and Mated Mothers of the L1 and T Lineages

Using only male eggs, we compared the egg size (width, length, and volume) of males produced by virgin and mated mothers in their whole lifespan and in the above-mentioned age groups. For this analysis, a one-way multivariate analysis of variance (MANOVA) was run with the factor of ‘mating status’.

### 2.7. Effect of Maternal Age on Egg Size

To investigate the effect of maternal age on egg size independently of the offspring sex, we used eggs produced by mated mothers of the L1 and T lineages. The age of the ovipositing mothers was recorded for each egg they laid until the mothers died. We modelled the egg volumes depending on the maternal age for their entire lifespan and age groups: 3–5 days, 7–10 days, and above 10 days. The maternal age effect on egg size in both genders of the L1 and T lineages was analyzed with linear regression models. The slopes of the trends were calculated and tested to see if they were significant.

### 2.8. Statistical Analysis

The statistical analyses were performed using IBM SPSS 25 [35]. The normality of the residuals was accepted by the absolute values of their skewness and kurtosis, as they were all below 1. Homogeneity of variance was tested with Levene’s test (*p* > 0.05). In the case of a significant overall MANOVA result, one-way between-subject effects were tested for all three variables (width, length, and volume) with Bonferroni’s adjustment.

## 3. Results

### 3.1. Effect of Mating and Egg Size on the Hatching Probability of the Eggs

In the L1 lineage, maternal mating status significantly influenced the probability of hatching. The overall number of oviposited eggs was 1305; 67% of 598 eggs and 44% of 707 eggs laid by virgin and mated mothers, respectively, hatched. Therefore, virgin mothers had a significantly higher probability of having eggs that hatched, whilst mated mothers had a significantly higher probability of having eggs that did not hatch (X^2^ = 68.962, df = 1, *p* < 0.001). In the T lineage, out of 2660 eggs, 1558 were oviposited by virgins and 1102 were oviposited by mated mothers. Mating status did not influence the probability of hatching (X^2^ = 2.098, df = 1, *p* = 0.147).

Moreover, egg size did not influence the probability of hatching in the virgin mothers of the L1 lineage (Wilk’s λ = 0.99, F (3:569) = 1.585, *p* = 0.19; see Table 1), whilst in the mated mothers of the L1 lineage, eggs that hatched were significantly larger than eggs that did not hatch (Wilk’s λ = 0.98, F (3:688) = 3.712, *p* < 0.05; see Table 1). For both virgin and mated mothers of the T lineage, egg size significantly influenced the probability of hatching. Eggs that hatched were significantly larger than eggs that did not hatch (Virgin: Wilk’s λ = 0.98, F (3:1529) = 9.19, *p* < 0.001; Mated: Wilk’s λ = 0.98, F (3:1080) = 7.083, *p* < 0.001; see Table 1). The significant differences were all manifested in the width and volume but not in the length of the eggs (width and volume, L1 mated: F (1:690) > 7.534, *p* < 0.05; T virgin: F (1:1531) > 12.716, *p* < 0.01; T mated: F (1:1082) > 6.710, *p* < 0.05; length, L1 mated: F (1:690) = 0.011, *p* = 0.92; T virgin: F (1:1531) = 5.171, *p* = 0.07; T mated: F (1:1082) = 0.977, *p* = 0.32).

### 3.2. Comparison of Male and Female Egg Size Produced by Mated Mothers of the L1 and T Lineages

Considering all the eggs measured throughout the entire lifespan of the mothers, there were no significant differences in the size of male and female eggs produced by mated mothers in either the L1 or T lineages (L1 lineage: Wilk’s λ = 0.97; F (3:202) = 2.291, *p* = 0.08; T lineage: Wilk’s λ = 0.98; F (3:428) = 2.528, *p* = 0.06; see Table 2).

There were also no significant differences in the size of male and female eggs produced by mated mothers of the L1 lineage at any given age group: (3–5-day-old mothers: Wilk’s λ = 0.95; F (3: 48) = 0.838, *p* = 0.48; 7–10-day-old mothers: Wilk’s λ = 0.98; F (3:60) = 0.441, *p* = 0.73; above-10-day-old mothers: Wilk’s λ = 0.94; F (3:44) = 0.916, *p* = 0.44; see Table 3). In the T lineage, the size of male and female eggs produced by mated mothers was not significantly different in the age groups of 3–5-day-old mothers and above-10-day-old mothers (3–5-day-old mothers: Wilk’s λ = 0.92; F (3:63) = 1.945, *p* = 0.13; above-10-day-old mothers: Wilk’s λ = 0.96; F (3:192) = 2.459, *p* = 0.06), while the difference was significant in the age group of 7–10 day-old-mothers (Wilk’s λ = 0.90; F (3:104) = 3.978, *p* < 0.05; see Table 3). According to the follow-up univariate analysis, at this age, only the width of female eggs was significantly larger than that of male eggs (Table 3).

### 3.3. Testing the Model of Egg Size and Sex Allocation

From all the eggs measured throughout the lifespan of the virgin and mated mothers of the L1 and T lineages, our results showed that eggs laid by mated mothers were significantly larger than eggs laid by virgin mothers in the L1 lineage (Wilk’s λ = 0.86, F (3:1301) = 72.523, *p* < 0.001; see Table 4). This was true for all three parameters of the egg size (F (3:1303) > 105.092, *p* < 0.001). In the T lineage, eggs laid by virgin mothers were significantly larger (Wilk’s λ = 0.98, F (3:2656) = 16.624, *p* < 0.001; see Table 4) in width and volume compared to the eggs laid by mated mothers for the entire lifespan (F (3:2658) > 11.980, *p* < 0.01), though with a significantly smaller length (F (3:2658) = 13.980, *p* < 0.001).

In the L1 lineage, eggs laid by mated mothers were significantly larger than eggs laid by virgin mothers in all maternal age groups (3–5-day-old mothers: Wilk’s λ = 0.80, F (3:312) = 25.958, *p* < 0.001; 7–10-day-old mothers: Wilk’s λ = 0.81, F (3:328) = 26.403, *p* < 0.001); and above-10-day-old mothers: Wilk’s λ = 0.95, F (3:420) = 7.654, *p* < 0.001; see Table 5). That was true for all three parameters of egg size (F (1:df) > 8.5, df = 314, 330 and 422, respectively; *p* < 0.01), except for the width of eggs laid by mothers older than 10 days, at which the difference in the size of eggs between virgin and mated mothers was not significant (F(1:422) = 1.873, *p* = 0.172). In the T lineage, eggs laid by mated mothers were significantly larger than eggs laid by virgin mothers in the maternal age group of 3–5-day-old mothers (Wilk’s λ = 0.96, F (3:388) = 5.926, *p* < 0.01; see Table 5), as detected in the width, length, and volume of the eggs (F (1:390) > 8.511, *p* < 0.01). Meanwhile, for the 7–10-day-old mothers, there was no significant difference in the size of eggs laid by virgin and mated mothers (Wilk’s λ = 0.99, F (3:555) = 2.315, *p* = 0.075; see Table 5), even though eggs produced by virgin mothers were slightly larger in width and volume than those produced by mated mothers. The difference was significant above 10 days of maternal age (Wilk’s λ = 0.96, F (3:1389) = 21.093, *p* < 0.001; see Table 5): eggs produced by virgin mothers were significantly larger than eggs produced by mated mothers for the width and egg volume, while eggs produced by mated mothers were significantly larger than those produced by virgin mothers, considering the length of the egg (F (1:1391) > 10.329, *p* < 0.01).

To summarize our results, we can state that the size distribution of the eggs of virgin and mated mothers compared within the L1 and within the T lineages were all normal with homogeneous variances, whether considering the entire lifespan of the mothers or the different age groups of the mothers. The only difference we found is in the mean values of the egg size distribution. Specifically, for the L1 lineage, the eggs of mated mothers were significantly larger (independently of which age group of the mothers was considered), and that for the T lineage, it seems that in the younger age group, rather the eggs of mated, in the older age group, the ones of virgin mothers were larger.

### 3.4. Comparison of Male Egg Size Produced by Virgin and Mated Mothers of the L1 and T Lineages

The size of male eggs produced by mated mothers was significantly larger than those produced by virgin mothers for the whole lifespan in the L1 lineage (Wilk’s λ = 0.93, F (3:680) = 18.261, *p* < 0.001; see Table 6). Highly significant differences were detected in all three parameters of the eggs: the width, length, and volume (F (1:682) > 22.200, *p* < 0.001). In the T lineage, there were no significant differences in the size of male eggs produced by virgin or mated mothers throughout the lifespan (Wilk’s λ = 0.99, F (3:1657) = 2.097, *p* = 0.10; see Table 6).

In the L1 lineage, the size of male eggs produced by mated mothers was significantly larger than that of produced by virgin mothers at the maternal age groups of 3–5-day-old and 7–10-day-old mothers (Wilk’s λ = 0.84, F (3:182) = 11.576, *p* < 0.001; Wilk’s λ = 0.86, F (3:130) = 7.298, *p* < 0.001, respectively; see Table 7). 

At maternal ages of above 10 days old, there were no significant differences in the size of male eggs produced by virgin and mated mothers (Wilk’s λ = 0.99, F (3:236) = 0.596, *p* = 0.62; see Table 7).

In the T lineage, there were no significant differences in the size of male eggs produced by virgin and mated mothers at the maternal age groups of 3–5-day-old and 7–10-day-old mothers (Wilk’s λ = 0.98, F (3:236) = 1.624, *p* = 0.19; Wilk’s λ = 0.98, F (3:355) = 2.423, *p* = 0.07; see Table 7). Moreover, at maternal age of above 10 days old, there were significant differences in male egg size produced by virgin and mated mothers (Wilk’s λ = 0.99, F (3:882) = 3.966, *p* < 0.01; see Table 7); considering both width and volume, eggs produced by virgin mothers were significantly larger than eggs produced by mated mothers.

### 3.5. Effect of Maternal Age on Egg Size

**Figure 3:**
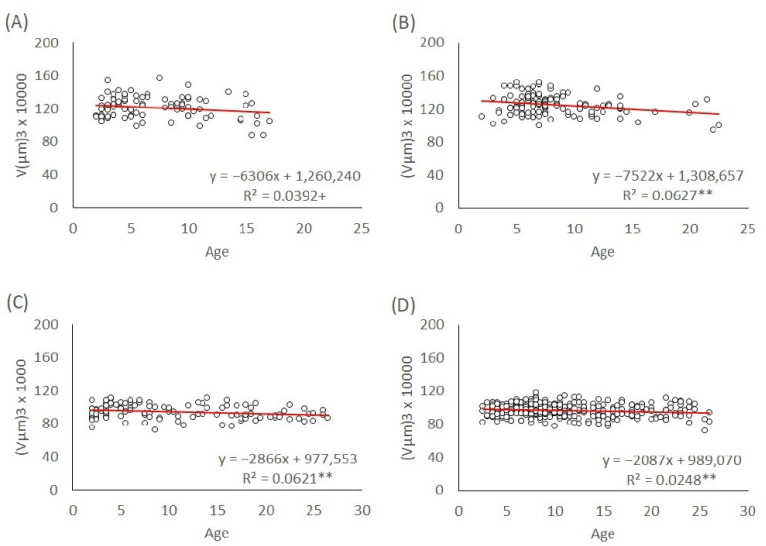
Linear regression of egg size throughout the lifespan of mothers. (**A**) L1 male eggs, (**B**) L1 female eggs, (**C**) T male eggs, and (**D**) T female eggs. The significance level of the slope: ** *p* < 0.01; + *p* < 0.1.

Egg volumes depending on maternal age are presented for the whole lifespan of the mothers in Figure 3. In the case of the L1 lineage, the trend of male egg volumes was slightly significantly decreasing with a slope s = −6306 (*p* = 0.06) (Figure 3A). For the L1 lineage, the trend of female egg volumes was also significantly decreasing with a slope s = −7522 (*p* < 0.01, Figure 3B). The male and female egg volumes of the T lineage are presented in Figure 3C,D, which shows that the trends were significantly decreasing (s = −2866, *p* < 0.01; s = −2087, *p* < 0.01, respectively).

**Figure 4:**
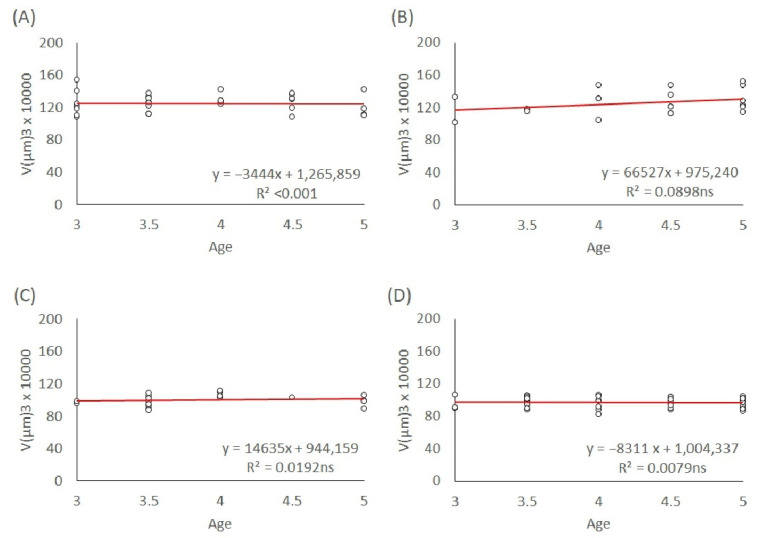
Linear regression of egg size in the maternal age group of 3–5-day-old mothers. (**A**) L1 male eggs, (**B**) L1 female eggs, (**C**) T male eggs, and (**D**) T female eggs. The significance level of the slope: ns (not significant).

Egg volumes depending on the maternal age of 3–5-day-old mothers are shown in Figure 4. In the case of the L1 lineage, the trends of male and female egg volumes were not significant (s = −34,434, *p* = 0.91; s = 66,527, *p* = 0.20, respectively; see Figure 4A,B). The male and female egg volumes of the T lineage are represented in Figure 4C,D, which shows that the trends were also not significant (s = 14,635, *p* = 0.56; s = −8311, *p* = 0.55, respectively).

**Figure 5:**
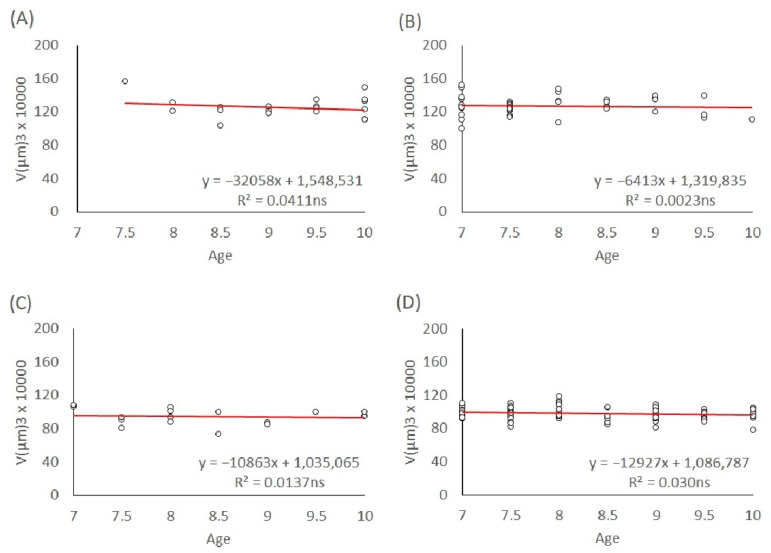
Linear regression of egg size in the maternal age group of 7–10-day-old mothers. (**A**) L1 male eggs, (**B**) L1 female eggs, (**C**) T male eggs, and (**D**) T female eggs. The significance level of the slope: ns (not significant).

Egg volumes depending on the maternal age of 7–10-day-old mothers are shown in Figure 5. For both the L1 and T lineages, the trends of male and female egg volumes were not significant (s = −32,058, *p* = 0.40; s = −64,123, *p* = 0.76; s = −10,863, *p* = 0.66; s = −129,267, *p* = 0.10, respectively; see Figure 5A–D).

**Figure 6:**
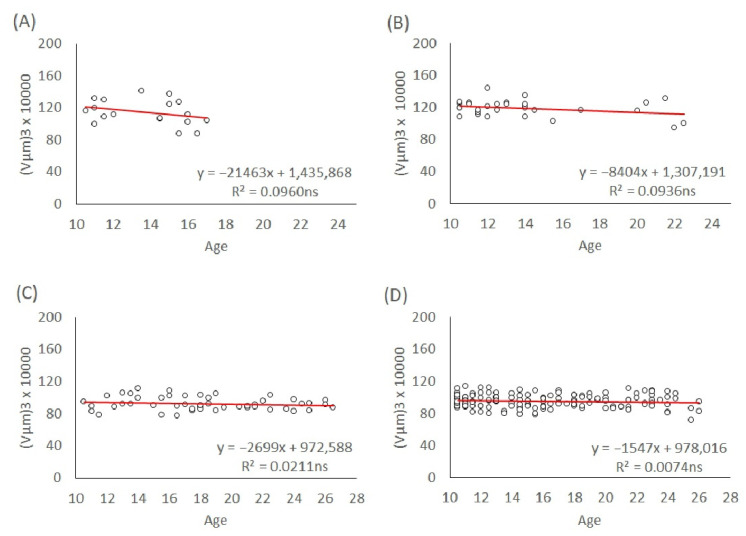
Linear regression of egg size in the maternal age groups above 10 days old. (**A**) L1 male eggs, (**B**) L1 female eggs, (**C**) T male eggs, and (**D**) T female eggs. The significance level of the slope: ns (not significant).

Egg volumes for the maternal age groups above 10 days are shown in Figure 6. The trends of male and female egg volumes were not significant in any of the L1 and T lineages (s = −21,463, *p* = 0.21; s = −8404, *p* = 0.100; s = −2699, *p* = 0.30; s = −1547, *p* = 0.30, respectively; see Figure 6A–D).

## 4. Discussion

The relationship between egg size and hatchability has already been studied for mites [21], but never in thrips. The fact that larger eggs are generally more likely to hatch and produce fast-developing juveniles with higher survivorship than smaller eggs in the spider mite *T. urticae* indicates that egg size might be a predictor of energy content in some cases [21], and our results confirm this: hatched eggs were significantly larger than the eggs that did not hatch in the L1 lineage laid by mated mothers and in the T lineage of *T. tabaci* for all eggs, independently of mating status. Mating also affected the hatching probability of the eggs in the L1 lineage, with more eggs hatching from virgins than from mated mothers, and mating did not affect the hatching probability of the eggs in the T lineage. In the bruchid beetle *Callosobruchus maculatus* F., mating (mated once or multiple times) had no detectable effect on the hatching probability of the eggs [36]. To our knowledge, no other study has addressed the effect of mating status on the hatching probability of the eggs.

Our key finding is that sex allocation in haplodiploid *T. tabaci* is not mediated via egg size like in the two-spotted spider mite *T. urticae* [21] and Kelly’s citrus thrips *P. kellyanus* [22]. In both species, egg size and gender were found to be related to the egg mass that was produced by young arrhenotokous females, 3–5-day-old mothers in *T. urticae* and 7–10-day-old mothers in *P. kellyanus*. The size of male and female eggs produced by mated mothers of the L1 lineage was not significantly different for the whole lifespan and at any age group, indicating that gender and egg size are independent of each other in this lineage regardless of maternal age. In the T lineage, however, gender only seemed to be dependent on egg size in the maternal age group of 7–10 days, but gender was independent of egg size in the progeny of younger and older mothers. Our results demonstrate that studying the relationship between egg size and gender in a narrow age group of mothers might lead to misconclusions unless there are different mechanisms working in mated females depending on their age.

By comparing the size of eggs produced by virgin and mated mothers, we showed that mated females laid significantly larger eggs throughout their lifespan and in all other age groups in the L1 lineage. The mean value of the egg size distribution of mated mothers in the L1 lineage was larger than that of virgins throughout their lifespan. This may suggest that there is no egg size that determines fertilization in this lineage—rather, it is the fertilization that may influence egg size. Eggs produced by mated mothers might receive more resources than those produced by virgins, and mating just increases egg size in general, thus leading to a larger size of male eggs produced by mated mothers than by virgins (Figure 2B, scenario 2). Contrary to the L1 lineage, in the T lineage, an entirely new scenario is needed to assess the obtained results; eggs laid throughout the lifespan of virgin mothers were significantly larger than those of mated mothers.

Our findings may indicate that these two lineages of *T. tabaci* have different resource allocation strategies in response to maternal mating status. In *P. kellyanus* [22], mating increases the early-life reproductive investment of females; therefore, mated females produce larger eggs than virgin females, just like our L1 lineage. However, the male offspring of mated mothers had smaller eggs than those of virgins [22]. In *Tetranychus ludeni* Zacher, virgin mothers laid significantly larger eggs than mated mothers, indicating a strategic resource allocation in response to mating status, with more resources being allocated to their male offspring when the mothers do not have the chance to produce female offspring [37]. Females may receive nutritional benefits during mating. Because males may provide nutrients to females with their ejaculate, mating is an activity of females that manipulates her nutritional status and therefore influences the size of her eggs. These nuptial gifts serve as paternal investment by increasing reproductive fitness (e.g., increasing egg size; Gwynne [38]). For the bruchid beetle *C. maculatus* F., multiple mating of a female has been proven to increase the size of eggs laid by her [36]. In haplodiploids, males can only pass their genes to female offspring, whereas the genes of females are passed on to both males and females. The effort of males to influence female production results in sexual conflict between males and females over the sex ratio of the progeny. During mating, males may transfer some seminal proteins that increase the sperm release from the spermatheca, which facilitates the fertilization rate [39]. A higher fertilization rate could also be achieved by increasing egg size after mating [40]. Moreover, if the selection of females has optimized egg size, females will use male nutrients to increase egg numbers. In contrast, male selection would favor increasing the size of the eggs he fertilizes rather than increasing the number of eggs [41]. This change in the egg size could be manipulated by shifting adaptations between early and late reproduction or between size and the number of offspring [42]. Further studies into sexual conflicts and sex allocation patterns need to be carried out in order to better understand the observed phenomenon in this cryptic species complex.

Maternal age influences the egg size in a wide group of insect species, including Coleoptera [36], Lepidoptera [43], Orthoptera [44], and Diptera [45]. According to studies of various authors, some data support the reduction of egg size over time with respect to maternal age [36,46]. Some other authors have observed an increase in egg size with increasing maternal age [47]. Some others have emphasized that there are no significant differences in egg size with respect to maternal age [48,49]. The most common explanation for this phenomenon is the resource depletion hypothesis, which suggests that it may be a physiological limitation of the females to produce offspring of the same size [36,50]. The observed decrease in egg size with increasing maternal age has been proven in two related species of bruchid beetles, *C. maculatus* F. and *Callosobruchus chinensis* Fab. (Coleoptera) [36,46]. It was found that females with diminished resources for egg production laid smaller eggs with increasing maternal age, and those who were allowed to access food laid larger eggs, even at later maternal ages. Alternatively, Begon and Parker [50] proposed that the decrease in egg size with increasing maternal age may be adaptive when the female clutch size is constrained. However, our experiment was not designed to test such a hypothesis.

In *T. tabaci*, maternal age had an overall effect on the egg size of both males and females in the L1 and T lineages throughout their lifespan. Egg size in both genders and lineages decreased with increasing maternal age. Because the maternal diet was not manipulated in this study, our results are not consistent with the resource depletion hypothesis. However, in the very young maternal age groups (3–5-day-old mothers, 7–10-day-old mothers, and older than 10 days), male and female egg size in both L1 and T lineages stayed at a constant level, without significant changes in egg size.

## 5. Conclusions

In conclusion, we did not detect egg-size-mediated sex allocation in the two known arrhenotokous lineages of *T. tabaci*. In the L1 lineage, egg size and gender were found to be independent of maternal age. In the T lineage, the egg size difference between males and females was only present in the maternal age group of 7–10 days. The hypothesis (Figure 2, scenario B) that mating increases the egg size was valid only for the L1 lineage, and an entirely new scenario is needed for the T lineage. Finally, the overall decreasing effect on egg size in both genders of the L1 and T lineages cannot be attributed to the resource depletion hypothesis. Our results provide the first empirical evidence that egg size has no influence on sex allocation and raises an intriguing need for the further testing of this process.

## Figures and Tables

**Table 1:** Size of hatched and unhatched eggs in the virgin and mated mothers of the L1 and T lineages.

Lineage	Mother Status	Hatchability	N	Width (μm)	Length (μm)	Volume (μm)^3^
L1	Virgin	Hatched	398	101.63 ± 5.23 a	210.79 ± 7.53 a	1,143,028 ± 122,622 a
		Did not hatch	200	100.14 ± 5.49 a	210.79 ± 8.54 a	1,110,542 ± 131,482 a
	Mated	Hatched	308	104.69 ± 4.56 b	215.82 ± 7.54 a	1,241,697 ± 124,026 b
		Did not hatch	399	103.92 ± 4.88 a	214.99 ± 8.55 a	1,218,441 ± 123,413 a
T	Virgin	Hatched	947	97.15 ± 3.77 b	196.19 ± 7.55 a	971,065 ± 86,207 b
		Did not hatch	611	96.39 ± 4.48 a	195.38 ± 8.52 a	953,078 ± 102,088 a
	Mated	Hatched	639	96.25 ± 3.64 b	197.11 ± 7.74 a	957,504 ± 81,936 b
		Did not hatch	463	95.48 ± 4.99 a	196.90 ± 7.74 a	942,762 ± 106,616 a

Data are presented as mean ± SD. N: number of tested individuals. Different letters are shown to indicate significant differences in egg size (*p* < 0.05).

**Table 2:** Size of male and female eggs produced by mated mothers of the L1 and T lineages throughout the lifespan.

Lineage	Gender	N	Width (μm)	Length (μm)	Volume (μm)^3^
L1	Male	91	103.68 ± 4.92	215.33 ± 8.24	1,215,783 ± 133,761
	Female	119	105.00 ± 4.31	215.25 ± 7.49	1,245,540 ± 120,556
T	Male	112	95.80 ± 3.60	196.50 ± 8.11	946,061 ± 85,649
	Female	325	96.61 ± 3.47	197.54 ± 7.77	966,454 ± 77,236

Data are presented as mean ± SD. N: number of tested individuals.

**Table 3:** Size of male and female eggs produced by mated mothers of the L1 and T lineages in different age groups.

Lineage	Age	Gender	N	Width (μm)	Length (μm)	Volume (μm)^3^
L1	3–5	Male	32	105.39 ± 4.50	215.00 ± 6.19	1,252,784 ± 115,861
		Female	20	105.75 ± 4.81	214.63 ± 8.24	1,261,308 ± 145,849
	7–10	Male	20	104.50 ± 4.56	218.75 ± 8.29	1,253,601 ± 126,684
		Female	44	105.51 ± 4.09	217.90 ± 7.55	1,272,476 ± 113,299
	>10	Male	18	101.94 ± 5.66	211.25 ± 7.63	1,153,668 ± 141,059
		Female	30	103.50 ± 4.08	211.75 ± 6.40	1,189,676 ± 101,846
T	3–5	Male	20	97.38 ± 4.01	199.00 ± 7.23	989,357 ± 85,396
		Female	47	96.38 ± 2.75	199.31 ± 7.78	969,857 ± 60,965
	7–10	Male	17	95.15 ± 3.59	198.53 ± 8.34	943,690 ± 94,740
		Female	91	97.69 ± 3.19 *	195.82 ± 7.67	979,679 ± 75,671
	>10	Male	50	95.20 ± 3.60	194.30 ± 8.69	923,248 ± 81,207
		Female	146	95.92 ± 3.69	197.53 ± 7.76	953,196 ± 83,045

Data are presented as mean ± SD. N: number of tested individuals. * is for significant gender difference in the width of the eggs (*p* < 0.05).

**Table 4:** Size of eggs produced by virgin and mated mothers of the L1 and T lineages throughout the lifespan.

Lineage	Mating Status	N	Width (μm)	Length (μm)	Volume (μm)^3^
L1	Virgin	598	101.13 ± 3.16 a	210.79 ± 7.87 a	1,132,163 ± 126,478 a
	Mated	707	104.26 ± 4.75 b	215.35 ± 8.13 b	1,228,572 ± 124,131 b
T	Virgin	1558	96.85 ± 4.08 b	195.87 ± 7.95 a	964,011 ± 93,143 b
	Mated	1102	95.92 ± 4.27 a	197.02 ± 7.74 b	951,310 ± 93,344 a

Data are presented as mean ± SD. N: number of tested individuals. Different letters are shown to indicate significant differences in egg size (*p* < 0.05).

**Table 5:** Size of eggs produced by virgin and mated mothers of the L1 and T lineages in different age groups.

Lineage	Age	Mating Status	N	Width (μm)	Length (μm)	Volume (μm)^3^
L1	3–5	Virgin	154	100.50 ± 5.31 a	211.85 ± 7.89 a	1,124,142 ± 128,114 a
		Mated	162	105.05 ± 4.29 b	215.15 ± 7.95 b	1,245,716 ± 117,415 b
	7–10	Virgin	114	102.48 ± 4.73 a	210.50 ± 8.35 a	1,159,649 ± 114,080 a
		Mated	218	105.02 ± 4.42 b	216.95 ± 7.81 b	1,254,857 ± 111,360 b
	>10	Virgin	222	101.80 ± 4.72 a	210.38 ± 7.42 a	1,144,270 ± 115,555 a
		Mated	202	102.45 ± 5.04 a	213.68 ± 8.03 b	1,177,647 ± 127,352 b
T	3–5	Virgin	220	95.33 ± 4.28 a	196.91 ± 7.55 a	939,622 ± 99,414 a
		Mated	172	96.60 ± 3.89 b	199.16 ± 7.59 b	974,596 ± 85,654 b
	7–10	Virgin	342	97.57 ± 3.98 a	197.22 ± 7.13 a	984,918 ± 88,818 a
		Mated	217	96.83 ± 3.73 a	196.46 ± 7.54 a	966,120 ± 83,611 a
	>10	Virgin	836	97.14 ± 3.89 b	194.91 ± 8.28 a	964,865 ± 90,767 b
		Mated	557	95.60 ± 4.41 a	196.33 ± 7.76 b	941,784 ± 96,684 a

Data are presented as mean ± SD. N: number of tested individuals. Different letters are shown to indicate significant differences in egg size (*p* < 0.05).

**Table 6:** Size of male eggs produced by virgin and mated mothers of the L1 and T lineages throughout the lifespan.

Lineage	Male Produced by	N	Width (μm)	Length (μm)	Volume (μm)^3^
L1	Virgin mother	597	101.21 ± 5.23 a	210.80 ± 7.87 a	1,133,672 ± 124,205 a
	Mated mother	91	103.68 ± 4.92 b	215.33 ± 8.24 b	1,215,783 ± 133,761 b
T	Virgin mother	1555	96.85 ± 4.06	195.90 ± 7.82	964,029 ± 91,621
	Mated mother	111	95.77 ± 3.60	196.82 ± 7.36	947,036 ± 85,410

Data are presented as mean ± SD. N: number of tested individuals. Different letters are shown to indicate significant differences in egg size (*p* < 0.05).

**Table 7:** Size of male eggs produced by virgin and mated mothers of the L1 and T lineages in different age groups.

Lineage	Age	Male Produced by	N	Width(μm)	Length (μm)	Volume (μm)^3^
L1	3–5	Virgin mother	154	100.50 ± 5.31 a	211.85 ± 7.89 a	1,124,142 ± 128,114 a
		Mated mother	32	105.39 ± 4.50 b	215.00 ± 6.19 b	1,252,784 ± 115,861 b
	7–10	Virgin mother	114	102.48 ± 4.73 a	210.50 ± 8.35 a	1,159,649 ± 114,080 a
		Mated mother	20	104.50 ± 4.56 b	218.75 ± 8.29 b	1,253,601 ± 126,684 b
	>10	Virgin mother	222	101.80 ± 4.72 a	210.38 ± 7.42 a	1,144,270 ± 115,555 a
		Mated mother	18	101.94 ± 5.66 a	211.25 ± 7.63 a	1,153,668 ± 141,059 a
T	3–5	Virgin mother	220	95.33 ± 4.28 a	196.91 ± 7.55 a	939,622 ± 99,414 a
		Mated mother	20	97.38 ± 4.01 a	199.00 ± 7.23 a	989,357 ± 85,396 a
	7–10	Virgin mother	342	97.57 ± 3.98 a	197.22 ± 7.13 a	984,918 ± 88,818 a
		Mated mother	17	95.15 ± 3.59 a	198.53 ± 8.34 a	943,690 ± 94,740 a
	>10	Virgin mother	836	97.14 ± 3.89 b	194.91 ± 8.28 a	964,865 ± 90,767 b
		Mated mother	50	95.20 ± 3.60 a	194.30 ± 8.69 a	923,248 ± 81,207 a

Data are presented as mean ± SD. N: number of tested individuals. Different letters are shown to indicate significant differences in egg size (*p* < 0.05).

## Data Availability

The data presented in this study are available upon request from the corresponding author.
